# Current Evidence and Practical Considerations for Adaptogen Use in Exercise Recovery, Training Adaptation, and Exercise Performance in Athletes: A Narrative Review

**DOI:** 10.3390/nu18152552

**Published:** 2026-08-04

**Authors:** Anna Książek

**Affiliations:** Department of Biological Principles of Physical Activity, Faculty of Physical Education and Sports, Wroclaw University of Health and Sport Sciences, 51-612 Wroclaw, Poland; anna.ksiazek@awf.wroc.pl; Tel.: +48-71-347-3363

**Keywords:** sports nutrition, dietary supplements, fatigue, *Withania somnifera*, *Rhodiola rosea*, *Panax*, *Cordyceps*

## Abstract

**Background/Objectives:** Exercise-induced muscle damage, inflammation, oxidative stress, and accumulated fatigue affect post-exercise recovery and long-term training adaptations in athletes. Consequently, nutritional strategies supporting recovery have gained attention in sports nutrition. Among emerging compounds, adaptogens have attracted particular interest due to their potential anti-fatigue, antioxidant, and sleep-enhancing properties. However, evidence in trained athletes remains limited. Therefore, the aim of this narrative review is to summarize the current scientific evidence regarding the use of selected adaptogens in sport and to evaluate their potential roles in exercise recovery, training adaptation and exercise performance. Particular attention is given to *Withania somnifera* (ashwagandha), *Panax* species, *Rhodiola rosea* and *Cordyceps* species. Furthermore, this review discusses the practical implications and current limitations of the available evidence for athletes and sports practitioners. **Conclusions:** Current evidence from a limited number of heterogeneous studies suggests that selected standardized adaptogen preparations may provide beneficial effects on specific exercise-recovery-related outcomes and selected aspects of exercise performance in trained athletes. Among the adaptogens discussed, most consistent evidence concerns *Withania somnifera*, whereas the evidence for *Panax* species, *Rhodiola rosea* and *Cordyceps* species remains limited and should be considered preliminary. Furthermore, the available literature is characterized by a limited number of studies conducted in trained athletes, the underrepresentation of female athletes, and considerable heterogeneity in extract composition and supplementation protocols. Consequently, the current evidence is insufficient to formulate definitive recommendations regarding the efficacy, optimal dosage, or long-term safety of adaptogen supplementation. Further well-designed randomized controlled trials are required to establish evidence-based recommendations.

## 1. Introduction

Dietary supplement use is common among athletes across different sports and performance levels, with studies indicating that approximately 40–90% of athletes report using dietary supplements [1,2,3]. The primary reasons for supplement use among athletes are to enhance muscle recovery and promote adaptation to training loads [4], which may consequently improve exercise performance. In addition to the most commonly used supplements, such as creatine, protein isolates, and caffeine [4], interest in plant-derived supplements has also increased. Data from McDaid and colleagues [5] indicate that a high proportion of athletes report using supplements regularly. Interestingly, this also includes herbal supplements, and about 16% of athletes reported using plant-derived products [5]. On the one hand, this is not a very high proportion, particularly considering that most adaptogens are classified in Group C according to the Australian Institute of Sport (AIS) supplement classification system (www.ais.gov.au, accessed on 4 July 2026). However, considering the growing interest in natural supplements, this trend is worth further attention.

Intense physical exercise and increasing training loads may induce considerable physiological stress, including exercise-induced muscle damage, inflammation, oxidative stress, and transient reductions in exercise performance [6]. Although these responses are considered a natural component of the training process, inadequate recovery may impair subsequent exercise performance and increase the risk of injury, overreaching or overtraining [7,8,9]. Therefore, effective recovery strategies are considered essential for restoring physiological and psychological function and supporting an athlete’s readiness for subsequent training sessions. Exercise recovery is a multifaceted process involving the restoration of physiological and psychological homeostasis, the resolution of fatigue, muscle repair and regeneration, and the normalization of inflammatory and oxidative stress responses [10,11]. Because effective recovery may facilitate subsequent exercise performance and support long-term training adaptations, nutritional and supplementation interventions are receiving increasing attention as potential strategies to support post-exercise recovery [12]. Among these, adaptogens have attracted particular interest due to their reported anti-inflammatory, antioxidant, stress-modulating, and anti-fatigue properties [13]. Nevertheless, it should be emphasized that most studies have been conducted either in young, healthy individuals or in patients with diagnosed medical conditions [14,15,16].

Although several recent reviews have discussed the effects of individual adaptogens on exercise performance or broader aspects of sports nutrition, the available evidence remains heterogeneous and fragmented. Moreover, important issues related to exercise recovery and to practical, safety, and regulatory considerations have received comparatively less attention. Considering the above, this narrative review focuses specifically on studies conducted in trained athletes. Therefore, the aim of this narrative review is to summarize the current scientific evidence regarding the use of selected adaptogens in sport and to evaluate their potential role in exercise recovery, training adaptation and exercise performance. Furthermore, this review aims to translate the available evidence into practical considerations for athletes and sports practitioners.

## 2. Methodological Approach

A literature review was conducted using the international databases PubMed, Web of Science, and Google Scholar to identify publications concerning the effects of adaptogens on post-exercise recovery, training adaptation, and selected aspects of athletes’ exercise performance. The final literature search was conducted on 3 July 2026. The search strategy was based on combinations of keywords connected by the Boolean operators AND and OR, including, among others: “adaptogens”, “plant adaptogens”, “herbal supplements”, “*Withania somnifera*”, “ashwagandha”, “*Panax ginseng*”, “*Panax notoginseng*”, “Ginseng”, “*Rhodiola rosea*”, “*Cordyceps*”, “*Cordyceps sinensis*”, “*Cordyceps militaris*”, “athletes”, “professional athletes”, “trained athletes”, “athletic population”, “recovery”, “muscle damage”, “delayed-onset muscle soreness”, “fatigue”, “oxidative stress”, “adaptation”, “inflammation”, “exercise”, “training”, “exercise performance”, “athletic performance”, and “sport supplements”. Publications were selected for inclusion in the review in a stepwise manner based on the analysis of titles, abstracts, and full-text articles. The synthesis of the available evidence was conducted in a narrative form, focusing on the comparison of study findings, the identification of areas of agreement and disagreement, and the evaluation of sources of heterogeneity, such as differences in adaptogen species, type of preparation, extract standardization, dose, supplementation duration, and the characteristics of the study populations. Four adaptogens (*Withania somnifera*, *Panax* species, *Rhodiola rosea*, and *Cordyceps* species) were selected for the present review because intervention studies conducted in athletes are available for these adaptogens, enabling the evaluation of their potential effects on exercise recovery, training adaptation, and exercise performance. Due to the narrative nature of this review, a formal systematic study selection procedure and risk of bias assessment were not performed. Therefore, the possibility of publication selection bias cannot be completely excluded.

The main evidence synthesis included articles published in English in peer-reviewed scientific journals. Priority was given to intervention studies conducted in trained athletes evaluating the effects of supplementation with *Withania somnifera*, *Panax* species, *Rhodiola rosea*, or *Cordyceps species* on parameters related to post-exercise recovery, training adaptation, and exercise performance. For the purpose of this review, trained athletes were defined as participants described in the original studies as competitive, sub-elite, elite, professional, or national-team athletes. Narrative reviews, systematic reviews, meta-analyses, and mechanistic studies were used as complementary sources for the discussion of mechanisms of action, safety, and the limitations of the available scientific evidence. Studies conducted exclusively in untrained, amateur or recreationally active individuals, animal studies, in vitro studies, case reports, and conference abstracts lacking a complete methodological description were excluded from the main evidence synthesis.

## 3. Adaptogens: Definition and Proposed Mechanisms of Action

Before discussing the potential applications of adaptogens in sport, it is appropriate to present their definition and the basic criteria for their classification. The term *adaptogen* was introduced in 1947 by the Soviet scientist Nikolai Lazarev, who described adaptogens as substances that increase the non-specific resistance of the body to stress [17]. However, plant-based adaptogens have been used in traditional medicine systems for centuries in different parts of the world, such as Ayurveda and Traditional Chinese Medicine, to enhance the body’s resistance to various stressors [18]. Later, in the 1960s, Brekhman and Dardymov further developed this concept and proposed three main criteria that a substance must meet to be classified as an adaptogen. Firstly, adaptogens should exhibit low toxicity, which means that they are considered safe and cause minimal disruption of physiological functions. Secondly, they should have non-specific stress-protective effects, thereby increasing resilience to multiple stressors. Thirdly, they should have a normalizing effect, which is related to the restoration of physiological balance [17]. It is also worth noting that in the 1970s, the first synthetic adaptogens were also developed. However, some of them later became problematic in sport. For example, bromantane was prohibited by the World Anti-Doping Agency (WADA) [19], and bemityl was included on the WADA Monitoring Program. Therefore, this review focuses primarily on natural adaptogens.

The exact mechanisms of action of adaptogens are still not fully understood. However, current evidence suggests that adaptogens may act through pleiotropic and multi-target modulation of the body’s stress response [18] to various stressors, including physical, chemical, biological, and psychological stresses [20]. It is believed that the effects of adaptogens are primarily mediated through modulation of one or both of the major systems responsible for the stress response: the hypothalamic–pituitary–adrenal (HPA) axis, which integrates the hormonal response with the activity of the sympathoadrenal system (SAS), and the SAS itself, which is responsible for the “fight-or-flight” response [21]. A key feature of adaptogen activity is the normalization of HPA axis function and the integration of immune, metabolic, and neuroendocrine responses, leading to the restoration of homeostasis under both acute and chronic stress conditions [18,20,22]. At the molecular level, adaptogens may modulate several signalling pathways involved in cellular stress response, energy metabolism, antioxidant defence, and mitochondrial function, including PI3K/Akt, AMPK/SIRT1/PGC-1α, and Nrf2/NF-κB [18,23,24,25]. An additional mechanism potentially involved in the long-term effects of adaptogens is the induction of heat shock proteins, particularly Hsp70 [18,20], as well as the activation of antioxidant pathways, including Nrf2-dependent responses [18,26]. These processes may support mitochondrial integrity, cellular homeostasis, and resistance to oxidative stress [18]. The mechanisms described may underlie the neuroprotective and anti-fatigue properties of adaptogens; however, it should be emphasized that most of the available evidence is derived from animal studies. Furthermore, evidence confirming these molecular mechanisms in humans remains limited, particularly in trained athletes; therefore, these proposed mechanisms should be interpreted with caution until validated in well-designed human studies.

Based on the mechanisms described above, adaptogens may contribute to improved exercise recovery and training adaptation in athletes. The proposed mechanisms may potentially contribute to maintaining homeostasis during intensive exercise, attenuating excessive physiological responses to exercise-induced stress, and facilitating post-exercise recovery processes. Nevertheless, it should be emphasized that the effects of adaptogens cannot be attributed to a single active compound but rather to the synergistic action of multiple bioactive constituents [23].

Several adaptogens have been investigated in the scientific literature. The most commonly studied include: *Withania somnifera*, *Panax* species, *Cordyceps* species, *Rhodiola rosea*, *Astragalus*, Holy Basil, *Schisandra chinensis*, and turmeric [13]. This review focuses on the four adaptogens for which relatively more evidence is available in athletic populations: *Withania somnifera*, *Panax* species, *Rhodiola rosea*, and *Cordyceps* species.

## 4. *Withania somnifera*

One of the most popular natural adaptogens in recent years is *Withania somnifera* (ashwagandha). Ashwagandha contains a complex mixture of bioactive compounds, among which withanolides, alkaloids, sitoindosides, and glycowithanolides are considered the most important. Withanolides, particularly withaferin A and withanolide D, are widely recognized as the main compounds responsible for the adaptogenic, anti-inflammatory, and antioxidant properties of this plant [27]. However, the contents of these compounds may vary considerably between preparations, which represents one of the major challenges in interpreting study findings and comparing the efficacy of different extracts.

**Figure 1:**
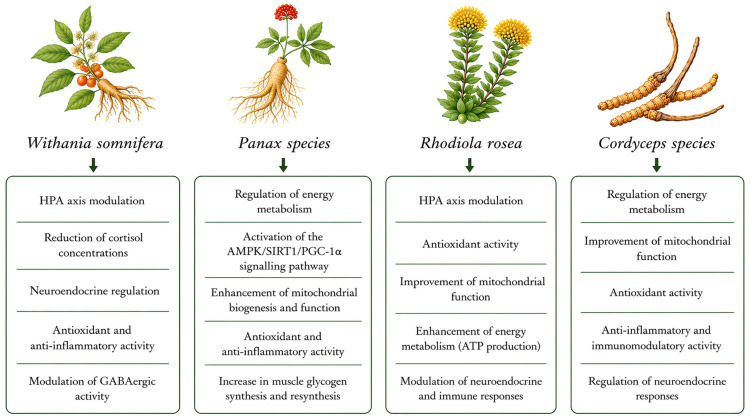
Proposed mechanisms of action of *Withania somnifera*, *Panax* species, *Rhodiola rosea*, and *Cordyceps* species. The figure summarizes the principal biological mechanisms proposed for these adaptogens based on the available literature. HPA: hypothalamic–pituitary–adrenal; AMPK: 5′AMP-activated protein kinase; SIRT1: sirtuin 1; PGC-1α: peroxisome proliferator-activated receptor gamma coactivator 1-alpha; ATP: adenosine triphosphate.

Several potential mechanisms of action of ashwagandha have been proposed, including modulation of the HPA axis and cortisol concentrations, as well as anti-inflammatory and antioxidant properties [28] (Figure 1). However, the effects of this adaptogen appear to be both direct and indirect. The direct mechanisms include the regulation of inflammatory processes, oxidative stress, and neuroendocrine function [29], whereas the indirect effects may result from improved sleep quality, enhanced GABAergic activity [30], reduced perceived stress [31] and beneficial effects on hormonal balance [29]. These mechanisms may potentially support exercise recovery processes and adaptation to training loads, which may explain the growing interest in ashwagandha supplementation in sport.

Studies investigating *Withania somnifera* supplementation suggest that this plant may have beneficial effects on both exercise performance [32] and selected parameters related to post-exercise recovery [33]. However, it should be emphasized that the vast majority of studies have been conducted in healthy individuals rather than trained athletes. The characteristic and main findings of intervention studies in athletes are summarized in Table 1.

Studies conducted in cyclists [34] and hockey players [35] demonstrated improvements in aerobic performance parameters, including VO_2max_ and time to exhaustion (TTE), as well as increased hemoglobin concentrations following 8 weeks of supplementation. These findings may suggest a beneficial effect of ashwagandha on selected parameters related to aerobic performance; however, the mechanisms underlying the observed changes require further investigation. At the same time, the number of available studies conducted in well-characterized athletic populations remains limited, and some studies included relatively small sample sizes. Particularly interesting are studies conducted in team sport athletes, in which greater emphasis was placed on parameters related to exercise recovery and the physiological response to exercise-induced stress. In a study conducted in professional female football players, supplementation with 600 mg/day of ashwagandha for 4 weeks improved the subjective perception of recovery (TQR) and sleep quality but had no effect on muscle strength or power parameters [36]. In turn, a study involving both female and male team sport athletes demonstrated partially different responses according to sex [37]. In women, supplementation was primarily associated with improvements in exercise recovery parameters, including the overall HI score, reductions in delayed onset muscle soreness (DOMS), and lower perceived fatigue, whereas in men, improvements were mainly observed in muscle power, as assessed by the countermovement jump (CMJ) [37]. These findings may suggest potential sex-related differences in the response to ashwagandha supplementation; however, the number of available studies involving female athletes remains very limited and requires further verification in larger athletic populations.

Based on the available evidence, ashwagandha appears to have the strongest support for improving selected recovery-related outcomes, including perceived recovery, sleep quality, DOMS and perceived fatigue. In contrast, improvements in aerobic performance, VO_2max_, hemoglobin concentration and muscle power should be interpreted as evidence of enhanced exercise performance or training adaptation. However, the available studies have mainly involved short-term interventions lasting between 4 and 8 weeks, with the most commonly used doses ranging from 600 to 1000 mg/day. Therefore, the effects of long-term supplementation on exercise recovery processes and training adaptations remain poorly understood. Further studies involving larger groups of athletes, including both women and athletes with different training status, are needed to determine the optimal duration and supplementation protocol and to verify whether the observed short-term benefits translate into long-term training adaptations.

## 5. *Panax*

The genus *Panax* comprises at least eleven species belonging to the family Araliaceae, among which *Panax ginseng* (Korean ginseng), *Panax quinquefolius* (American ginseng), and *Panax notoginseng* (notoginseng) are of the greatest scientific and practical importances. Although these plants are commonly referred to collectively as “ginseng”, they differ in their phytochemical profiles and potential biological activities [38]. The main bioactive compounds of the genus *Panax* are ginsenosides; however, their qualitative and quantitative composition depends on the species [39]. For example, ginsenoside Rf is considered a characteristic compound of *P. ginseng*, whereas pseudoginsenoside F11 is found mainly in *P. quinquefolius*. In contrast, *P. notoginseng* is characterized by the presence of notoginsenoside R1 and a high total saponin content [38]. In addition to species-related differences, the method of raw material processing (e.g., white ginseng, red ginseng, black ginseng), plant age, the plant part used, and the extraction method are also important, as they may substantially alter the ginsenoside profile and biological activity of the preparation [39,40]. These factors should be taken into account when interpreting the findings of studies investigating supplementation with *Panax* preparations in athletes.

Several potential mechanisms of action of *Panax* preparations have been proposed, primarily including the regulation of energy metabolism [41], as well as anti-inflammatory and antioxidant properties [42,43] (Figure 1). However, the effects of these adaptogens appear to be multifaceted, involving both direct effects on skeletal muscle cell function and indirect modulation of the physiological response to exercise-induced stress [43]. The direct mechanisms include activation of the AMPK/SIRT1/PGC-1α signalling pathway [43], leading to enhanced mitochondrial biogenesis, improved oxidative muscle function [44] and increased muscle glycogen synthesis and resynthesis [45], which may contribute to greater energy availability during exercise and post-exercise recovery. Furthermore, ginsenosides have been shown to reduce oxidative stress and the inflammatory response through modulation of the NF-κB and MAPK signalling pathways [43,46], leading to reduced production of reactive oxygen species and pro-inflammatory cytokines [43]. The indirect effects of supplementation may result from enhanced resistance to fatigue and beneficial effects on post-exercise recovery following intensive exercise [43]. These mechanisms may support adaptation to training loads and muscle recovery; however, as mentioned previously, most of the available evidence regarding the underlying molecular mechanisms is derived from preclinical studies.

The available studies investigating supplementation with *Panax* preparations in athletes are relatively limited and are characterized by substantial heterogeneity with regard to the species used, the type of extract, supplementation duration, and the exercise and recovery-related parameters evaluated (Table 2). Most of the available evidence concerns *Panax ginseng* [47,48], whereas only one study investigated the effects of *Panax notoginseng* [49], which further complicates direct comparison of the findings. Despite these limitations, the available studies suggest that *Panax* supplementation may influence both post-exercise recovery and exercise-related physiological responses. However, the evidence for these outcomes is not equivalent, as some studies evaluated direct recovery measures, whereas others focused primarily on metabolic responses or training adaptations. In the study by Pumpa et al. [49], supplementation with *Panax notoginseng* in an eccentric exercise model inducing DOMS did not significantly affect most markers of muscle damage and inflammation, leading the authors to conclude that *Panax notoginseng* supplementation had no substantial effect on recovery following eccentric exercise. However, a partial attenuation of the decline in functional performance and muscle soreness after exercise was observed [49]. In turn, Cristina-Souza et al. [48] demonstrated that short-term supplementation with *Panax ginseng* may increase neuromuscular activation and accelerate the recovery of muscle strength following eccentric exercise, despite the absence of changes in the classical markers of muscle damage, such as creatine kinase (CK) and lactate dehydrogenase (LDH) [48]. These findings may suggest that the potential effects of *Panax* preparations are more closely related to the modulation of neuromuscular function and exercise perception than to a direct influence on muscle damage. Different aspects of the effects of *Panax* preparations were investigated in studies evaluating the metabolic response to exercise. Similarly, Yan et al. [47] observed reductions in exercise-induced fatigue markers, together with beneficial effects on the metabolic profile of professional rowers during an intensive training period [47]. These findings primarily reflect physiological and metabolic adaptations to training rather than direct post-exercise recovery outcomes. Although such adaptations may indirectly support recovery capacity, they should not be interpreted as direct evidence of improved recovery. Nevertheless, it should be emphasized that the available studies include relatively small groups of athletes, frequently use different preparations, and differ with respect to extract standardization and supplementation protocols. This limits the possibility of drawing definitive conclusions regarding the efficacy of *Panax* supplementation in sport and highlights the need for further well-controlled studies conducted in trained athletic populations.

Based on the available evidence, *Panax* preparations may support selected aspects of post-exercise recovery, particularly neuromuscular recovery following eccentric exercise and reductions in perceived exertion. In contrast, most of the remaining evidence concerns metabolic responses to exercise or training adaptations rather than direct measures of post-exercise recovery. However, the available studies have been limited to short-term supplementation and are characterized by substantial heterogeneity with regard to the *Panax* species, extract type, and supplementation protocols. Therefore, the current state of knowledge does not allow identification of the optimal preparation or supplementation protocol for athletes.

## 6. *Rhodiola rosea*

*Rhodiola rosea* is a traditional perennial medicinal plant that grows in rocky crevices and coastal cliffs of North America, the Arctic regions of Europe, and Asia. It belongs to the family *Crassulaceae* and has been used for medicinal purposes for centuries [50]. The adaptogenic effects of *Rhodiola rosea* are primarily attributed to the bioactive compounds present in its root, among which salidroside and rosavins are considered the most important [51]. However, it should be emphasized that at least 109 chemical compounds have been identified in *Rhodiola rosea* [52]. Available evidence indicates that the bioactive constituents of *Rhodiola rosea* may exert anti-stress and anti-fatigue effects [53,54].

Several potential mechanisms have been proposed to explain the effects of *Rhodiola rosea*, primarily involving adaptogenic, antioxidant, and anti-fatigue properties [18,55] (Figure 1). As mentioned previously, salidroside and rosavins are considered the principal bioactive compounds, which, through modulation of the HPA axis and the PI3K/Akt [25,56], Nrf2 [26] and SIRT1 signalling pathways [18], may reduce oxidative stress, improve mitochondrial function, and enhance cellular resistance to stress. At the same time, available evidence indicates that *Rhodiola rosea* supplementation may influence energy metabolism by increasing ATP production and improving mitochondrial function [57], as well as modulating the immune [58] and neuroendocrine responses under conditions of increased physical and psychological stress [18]. These mechanisms may contribute to reduced fatigue perception and attenuation of oxidative stress-induced damage [18,55], which may provide a biological rationale for the growing interest in *Rhodiola rosea* among athletes. Nevertheless, similar to the previously discussed adaptogens, it should be emphasized that most of the proposed mechanisms of action of *Rhodiola rosea* are derived from in vitro and animal studies, whereas the number of studies confirming their relevance in trained athletes remains limited.

The available studies investigating *Rhodiola rosea* supplementation in athletes have yielded inconsistent findings regarding both post-exercise recovery and exercise performance (Table 3). While several studies evaluated outcomes directly related to recovery, others primarily assessed exercise performance or physiological responses to exercise, making direct comparisons difficult. Studies conducted in rowers [59] and marathon runners [60] did not demonstrate significant effects of *Rhodiola rosea* supplementation on exercise performance, exercise duration, VO_2max_, or indirect markers of muscle damage. Some studies, however, reported reductions in post-exercise lactate concentrations and markers of muscle damage [61,62], suggesting that *Rhodiola rosea* may influence the physiological response to exercise despite the absence of direct improvements in exercise performance. In the study by Shanely et al. [60], *Rhodiola rosea* supplementation had no effect on marathon performance, DOMS, markers of muscle damage, inflammatory response, or eHSP72 concentrations following marathon running, indicating limited efficacy during prolonged endurance exercise [60]. In contrast, more recent studies conducted in team sport athletes have demonstrated more promising effects. In basketball players, *Rhodiola rosea* supplementation improved aerobic performance, reduced perceived fatigue, and favourably affected performance during a simulated game [63]. Similar observations were reported in football players [62], in whom supplementation improved the ability to perform repeated high-intensity exercise, reduced post-exercise lactate concentrations, and positively affected decision-making and the maintenance of performance under fatigue. These findings suggest that *Rhodiola rosea* may enhance exercise performance during repeated high-intensity efforts and reduce perceived fatigue. However, these outcomes primarily reflect exercise performance and fatigue tolerance rather than direct measures of post-exercise recovery. Nevertheless, the considerable heterogeneity of supplementation protocols, doses, intervention duration, and outcome measures complicates the interpretation of the available evidence and highlights the need for further studies conducted in well-characterized athletic populations.

Based on the available evidence, *Rhodiola rosea* supplementation may beneficially affect selected parameters related to exercise-induced fatigue, perceived exertion, and recovery following intense physical exercise. However, the current state of knowledge does not allow definitive conclusions regarding its effects on exercise performance or the identification of an optimal supplementation protocol for athletes.

## 7. *Cordyceps*

Unlike the previously discussed plant-derived adaptogens, *Cordyceps* is a genus of fungi that grows on insect larvae and is attributed with adaptogenic properties. More than 350 species associated with *Cordyceps* have been identified worldwide; however, *Cordyceps sinensis* (*Ophiocordyceps sinensis*) and *Cordyceps militaris* are considered the two species of greatest therapeutic importance [64]. *Cordyceps sinensis* occurs naturally on the Tibetan Plateau and has been used in traditional medicine for centuries. However, its limited availability and high cost have led to increasing interest in *Cordyceps militaris*, which can be cultivated under controlled conditions [65]. Both species exhibit similar biological activities, although *Cordyceps militaris* generally contains higher concentrations of cordycepin, which is regarded as one of its principal bioactive compounds [66]. It should also be noted that *Cordyceps sinensis* is available in both wild and cultivated forms. Although they share a similar qualitative profile of the main bioactive compounds, differences in their concentrations have been reported. Cultivated *Cordyceps sinensis* has been shown to contain higher levels of adenosine and cordycepin, but lower levels of mannitol compared with the wild form, which may affect the comparability of preparations and the interpretation of study findings [67]. In addition to cordycepin, adenosine, and mannitol, the major bioactive compounds identified in *Cordyceps* species include polysaccharides, sterols, and cordycepic acid [64,68], which are attributed with antioxidant, immunomodulatory, and energy-metabolism-supporting properties [69].

Several potential mechanisms of action have been proposed for *Cordyceps*, including antioxidant, anti-inflammatory, and immunomodulatory effects [64,65,68,70], as well as regulation of the neuroendocrine response to stress and energy metabolism [71] (Figure 1). The effects of *Cordyceps* preparations are thought to result from the simultaneous modulation of multiple signalling pathways involved in the maintenance of cellular homeostasis, mitochondrial function, inflammatory processes, and adaptation to stress [72,73,74]. These mechanisms may potentially support recovery processes, enhance resistance to fatigue, and facilitate adaptation to increased training loads, which may explain the growing interest in *Cordyceps* supplementation in sport [64].

The available studies investigating *Cordyceps* supplementation in athletes are limited and have primarily involved endurance-trained athletes (Table 4).

Current evidence suggests that studies of *Cordyceps* supplementation have primarily evaluated exercise performance and training adaptations rather than direct measures of post-exercise recovery. Some studies reported improvements in selected parameters of aerobic performance, including VO_2max_, time to exhaustion (TTE), and ventilatory threshold (VT) [76]; however, these effects have not been consistently observed across studies [75]. Importantly, some studies also evaluated indirect markers of muscle damage and physiological adaptations to prolonged training loads, in addition to exercise outcomes. In the study by Nakamura et al. [77], supplementation with *Cordyceps militaris* was associated with attenuation of the increase in creatine kinase (CK) concentrations and favorable effects on selected hematological and iron metabolism parameters. However, no improvement was observed in 5 km running performance, suggesting that the potential benefits of supplementation may be related primarily to exercise recovery and adaptive processes rather than direct improvements in exercise performance [77]. A similar pattern was reported in a study involving trained female football players, in which four weeks of *Cordyceps sinensis* supplementation combined with repeated sprint training under normoxic or hypoxic conditions increased TTE and improved selected parameters of repeated sprint ability and fatigue index. At the same time, no changes were observed in VO_2max_ or hematological parameters, suggesting that the observed effects may be more closely related to improved tolerance to high-intensity exercise and training adaptations than to enhanced oxygen transport capacity [78]. These findings therefore appear to reflect training adaptation and fatigue tolerance rather than direct post-exercise recovery outcomes. Collectively, the available evidence suggests that *Cordyceps* supplementation may influence both exercise performance and training adaptation. However, direct evidence supporting improvements in post-exercise recovery remains limited and is largely restricted to indirect markers, such as exercise-induced muscle damage or fatigue-related responses, rather than functional measures of exercise recovery.

It should be emphasized that the available studies include relatively small groups of athletes and have investigated two different fungal species (*Cordyceps sinensis* and *Cordyceps militaris*), which differ in their bioactive compound profiles, as well as different preparations, doses, and short-term supplementation protocols. This limits the possibility of drawing definitive conclusions regarding the efficacy of *Cordyceps* supplementation in sport and highlights the need for further well-controlled studies conducted in trained athletic populations.

## 8. Regulation and Safety

Regardless of the potential efficacy of adaptogens, the safety of supplementation and the quality of the preparations used remain equally important considerations. Herbal adaptogens are typically marketed as dietary supplements rather than medicinal products. In most European Union Member States, dietary supplements are regulated as food products under EU food law [79], which means they do not require the same level of clinical testing as pharmaceutical drugs before entering the market. Therefore, athletes should be aware of the potential risks associated with contamination, the lack of extract standardization, and variability in supplement quality. Increasing evidence indicates that plant-derived dietary supplements may be characterized by considerable variability in composition and discrepancies between the declared and actual contents of active ingredient [80], as well as contamination with heavy metals [81], pesticides, and undeclared biologically active substances [82]. Furthermore, the quality of dietary supplements has been shown to vary not only between manufacturers but also between batches of the same product, which may substantially affect both the safety and efficacy of supplementation [82].

According to the 2026 WADA Prohibited List and Monitoring Program, the adaptogens discussed in this review are not included as prohibited substances. However, the fact that an adaptogenic ingredient is not prohibited does not guarantee that a commercially available dietary supplement containing that ingredient is free from prohibited substances or manufacturing-related contamination. Therefore, the anti-doping risk associated with adaptogen supplementation is primarily related to contamination, adulteration, and mislabelling of dietary supplements rather than to the adaptogenic ingredient themselves. It should be noted that contaminated dietary supplements account for approximately 6.4% to 26% of all anti-doping rule violations [83,84].

Although most clinical studies indicate that adaptogen supplementation is generally well-tolerated, the quality-related issues described above should be taken into consideration, as they have a substantial impact on the safety of plant-derived supplements. Liang et al. [85] conducted a scoping review on the safety of natural products, including those with adaptogenic properties, together with an analysis of adverse event reports from the WHO-UMC VigiBase database. A total of 479 publications and 45,042 adverse event reports related to natural products were included in the analysis. The most frequently reported adverse events involved gastrointestinal disorders, skin reactions, hepatotoxicity, cardiovascular disorders, and immune-related reactions. The authors also highlighted the potential for interactions between adaptogens and medications, as well as the lack of extract standardization and the insufficient reporting of adverse events in clinical studies [85].

Across the available clinical studies, reported adverse events have generally been mild and transient, most commonly including gastrointestinal complaints (e.g., nausea, diarrhea, constipation), dry mouth, reduced appetite, and occasional upper respiratory symptoms [13,86]. Among the adaptogens discussed in this review, the greatest amount of clinical safety data is available for *Withania somnifera* [28,29,87]. Nevertheless, reports of rare adverse events, potential herb–drug interactions, and variability in the composition of commercially available preparations highlight the importance of using standardized, high-quality products [85,87]. In contrast, safety data for *Rhodiola rosea*, *Panax* species, and *Cordyceps* species remain limited, particularly in athletic populations, and the current evidence is insufficient to draw firm conclusions regarding their long-term safety [85,88]. Furthermore, the absence of adverse-event reporting in individual clinical trials should not be interpreted as evidence of long-term safety, because most intervention studies were of relatively short duration and were not specifically designed to systematically evaluate adverse events or safety outcomes [13].

Another issue worth discussing concerns the growing concerns regarding the safety of ashwagandha supplementation that have emerged in recent years. In 2023, the Danish Veterinary and Food Administration concluded that products containing ashwagandha cannot be legally marketed as foods in Denmark because of identified safety concerns related to reproductive, hormonal, and neurological effects. However, this decision has been criticized by some researchers [89], who pointed to methodological limitations of the underlying risk assessment report and emphasized the lack of consistent clinical evidence demonstrating serious adverse effects associated with standardized Ashwagandha root extracts. At the same time, many clinical studies in humans have reported a favorable safety profile when standardized ashwagandha preparations are used at the recommended doses, without clinically significant changes in hematological, biochemical, or thyroid function parameters [28,29], whereas the reported adverse events were generally mild and transient in nature [87]. Considering the above, and given the limited available evidence, variability in supplement composition, and the lack of extract standardization, a causal relationship remains unclear. Therefore, further studies are needed to evaluate the long-term safety of ashwagandha supplementation, particularly at higher doses.

With the growing popularity of adaptogens among athletes, ensuring the quality and safety of the preparations used has become increasingly important. At the same time, more rigorous quality control and transparent product labeling are needed to promote the safer use of adaptogens and facilitate the interpretation of findings from future scientific studies. Future studies should also incorporate standardized and systematic adverse-event reporting to improve the quality of safety evidence for adaptogen supplementation in athletes.

## 9. Practical Considerations for Athletes

Despite the growing popularity of adaptogens in sport, the decision to use these supplements should be preceded by an assessment of the actual need for supplementation and a careful evaluation of the potential benefits and risks. The findings of our previous study demonstrated that dietary supplements are widely used among athletes; however, awareness of independent quality certification programs remains very low, even among professional athletes [2]. These findings highlight the need for educational initiatives aimed at promoting safe supplementation practices and increasing awareness of products that undergo independent quality testing.

From a practical perspective, athletes should choose dietary supplements certified by independent quality assurance programs, such as Informed Sport, NSF Certified for Sport, BSCG Certified Drug Free, HASTA, and the Cologne List, which reduce the risk of contamination with prohibited substances and minimize the likelihood of unintentional anti-doping rule violations.

Based on the current evidence, routine supplementation with adaptogens cannot currently be recommended for athletes due to the limited number of available studies and their considerable heterogeneity. A summary of the potential applications, practical considerations, current evidence, and main limitations for each adaptogen is provided in Table 5. Although some studies have reported potentially beneficial effects of selected adaptogens on specific exercise-recovery-, exercise-performance-, or training-adaptation-related outcomes, the available evidence is not sufficiently consistent to support their routine use. This position is consistent with the recommendations of the Australian Institute of Sport, which classifies adaptogens as Group C supplements, indicating that the current evidence does not support their routine use in athlete supplementation programs. Importantly, although improved exercise recovery may facilitate subsequent training quality and exercise performance, recovery–support interventions [10] should not automatically be assumed to enhance long-term training adaptations. Exercise-induced oxidative and inflammatory signalling play important roles in adaptive processes [90], and although several adaptogens exhibit antioxidant and anti-inflammatory properties, it remains unclear whether these mechanisms enhance, attenuate, or have no meaningful influence on long-term training adaptations in trained athletes. Thus, future studies should evaluate both short-term recovery outcomes and long-term physiological adaptations to repeated training stimuli.

Therefore, the decision to use adaptogens should be made on an individual basis under the supervision of a qualified sports dietitian or physician, taking into account the athlete’s health status, sport-specific demands, training phase, and supplementation goals. Furthermore, only standardized products from reputable manufacturers that have undergone independent batch testing should be considered. Adaptogens should not replace the fundamental pillars of exercise recovery, training adaptation, and exercise performance, including adequate energy and nutrient intake, a well-designed training program, sufficient sleep quantity and quality, and evidence-based recovery strategies.

## 10. Conclusions

The available scientific evidence suggests that selected standardized adaptogen preparations may beneficially affect recovery-related, training adaptation and selected parameters of exercise performance in athletes. Among the adaptogens discussed, *Withania somnifera* is currently supported by the most consistent evidence for selected outcome measures across the available studies in athletic populations. In contrast, the evidence regarding *Rhodiola rosea*, *Panax* preparations, and *Cordyceps* preparations remains promising but preliminary, owing to the smaller number of studies and greater heterogeneity of the available findings.

The interpretation of the available evidence is hampered by the limited number of studies conducted in trained athletes, generally small sample sizes, relatively short intervention periods, the underrepresentation of female athletes and the lack of sex-specific analyses, as well as the substantial heterogeneity in the species investigated, extract standardization, and supplementation protocols. Consequently, the current state of knowledge does not allow definitive recommendations regarding the optimal preparation, dosage, or duration of supplementation.

In conclusion, selected adaptogens represent a promising area of research in the context of supporting exercise recovery and training adaptation in athletes. However, the available evidence remains preliminary, outcome-specific and requires confirmation in well-designed randomized controlled trials conducted in well-characterized populations of trained athletes.

## Figures and Tables

**Table 1:** Effects of *Withania somnifera* (Ashwagandha) supplementation on exercise recovery, exercise performance, and selected physiological responses in athletes.

Authors	Study Group	Study Design	Intervention	Outcome	Conclusion
Shenoy et al. [34]	37 (20 F, 17 M) elite cyclists	Randomized, placebo-controlled clinical trial; 8 weeks	Standardized aqueous root extract; 1000 mg/day Incremental treadmill test (Bruce protocol)	↑ VO_2max_ ↑ TTE ↔ RER	*WS* improved selected indices of cardiorespiratory endurance of elite athletes
Malik et al. [35]	32 M hockey players	Randomized, single-blind, placebo-controlled clinical trial; 8 weeks	Standardized aqueous root extract; 1000 mg/day Cooper 12 min running test	↑ VO_2max_ ↑ Hb	*WS* supplementation improved VO_2max_ and Hb levels in the experimental group
Coope et al. [36]	30 F professional football players	Randomized, double-blind, placebo-controlled clinical trial; 4 weeks	Standardized root extract (KSM-66^®^, >5% withanolides); 600 mg/day Regular football training	↑ TQR ↑ Sleep quality ↔ Strength/power	*WS* supplementation improved exercise recovery and sleep quality, but did not affect strength and power performance in female footballers
Coope et al. [37]	56 (28 F, 28 M) sub-elite team-sport athletes	Randomized, double-blind, placebo-controlled clinical trial; 6 weeks	Standardized root extract (KSM-66^®^, >5% withanolides); 600 mg/day Pre-season training programme	↓ Cortisol ↓ Cortisone ↓ DOMS ↓ HI score ↓ Fatigue perception ↑ CMJ	*WS* supplementation may stabilise stress biomarkers, improve recovery perception and enhance muscle performance during pre-season training

F: female; M: male; *WS*: *Withania somnifera*; VO_2max:_ maximal oxygen uptake; TTE: time-to-exhaustion; RER: respiratory exchange ratio; Hb: hemoglobin; TQR: total quality recovery; DOMS: delayed onset muscle soreness; HI: Hooper Index; CMJ: countermovement jump; ↑/↓: statistically significant higher/lower values or changes in the supplementation group versus placebo/control, including significant group × time interactions; where no between-group comparison was reported, arrows indicate significant pre–post increases/decreases within the supplementation group; ↔: no statistically significant between-group effect. Arrows indicate direction, not benefit.

**Table 2:** Effects of *Panax* species supplementation on exercise recovery, exercise performance, and selected physiological responses in athletes.

Authors	Study Group	Study Design	Intervention	Outcome	Conclusion
Pumpa et al. [49]	20 M well-trained athletes	Randomized, double-blind, placebo-controlled clinical trial; acute supplementation (96 h)	*PN*: capsules; 4 g pre- and post-exercise + repeated doses for 96 h Downhill treadmill run (5 × 8 min, −10%, 80% HRmax)	↓ SJ (immediately post-exercise) ↑ Quadriceps pain (96 h) ↔ CMJ, CK, myoglobin, CRP	*PN* did not improve exercise recovery or performance in trained athletes
Yan et al. [47]	21 M professional rowers	Randomized, placebo-controlled clinical trial; 30 days	*PG*: powdered dried whole root (7-year-old Korean ginseng); 5 g/day Intensive rowing training period	↓ CK ↓ BUN ↑ T	*PG* supplementation favorably modulated biochemical markers associated with intensive training in the experimental group following the 30-day training period
Cristina-Souza et al. [48]	10 M competitive track and field athletes (adolescents)	Randomized, double-blind, crossover clinical trial; 8 days	*PG*: standardized oven-dried root (10% ginsenosides); 100 mg/kg BW/day Eccentric half-squat protocol	↓ RPE ↑ EMG activity ↑ MIVC recovery (24 h vs. PL; 48 h vs. PL) ↔ LDH, CK, DOMS	*PG* increased muscle recruitment, reduced perceived effort and accelerated recovery of muscle force after eccentric exercise

M: male; *PN*: *Panax notoginseng*; *PG*: *Panax ginseng*; BW: body weight; SJ: squat jump; CMJ: countermovement jump; CK: creatine kinase; CRP: C-reactive protein; BUN: blood urea nitrogen; T: testosterone; RPE: rating of perceived exertion; EMG: electromyography; MIVC: maximal isometric voluntary contraction; LDH: lactate dehydrogenase; DOMS: delayed onset muscle soreness; VO_2max:_ maximal oxygen uptake; PL: placebo; ↑/↓: statistically significant higher/lower values or changes in the supplementation group versus placebo/control, including significant group × time interactions; where no between-group comparison was reported, arrows indicate significant pre–post increases/decreases within the supplementation group; ↔: no statistically significant between-group effect. Arrows indicate direction, not benefit.

**Table 3:** Effects of *Rhodiola rosea* supplementation on exercise recovery, exercise performance, and selected physiological responses in athletes.

Authors	Study Group	Study Design	Intervention	Outcome	Conclusion
Skarpańska-Stejnborn et al. [59]	22 M professional rowers (Polish Rowing National Team)	Randomized, double-blind, placebo-controlled clinical trial; 4 weeks	*RR*: Rhodiolin^®^ (*Rhodiola rosea* extract); 200 mg/day Rowing ergometer (2000 m max test)	↔ Power output ↔ Exercise time ↔ CK, blood lactate ↑ TAC ↓ SOD activity immediately and 24 h post-exercise	*RR* increased plasma TAC and was associated with lower post-exercise SOD activity but did not affect rowing performance, or CK
Parisi et al. [61]	14 M well-trained endurance athletes	Randomized, double-blind, placebo-controlled clinical trial; 4 weeks	*RR*: commercial preparation; 170 mg/day Bicycle ergometer (cycle to exhaustion at 75% VO_2max_)	↔ VO_2max_, HR, RPE, TTE ↓ Lactate ↓ CK	*RR* reduced lactate and muscle damage markers but did not improve exercise performance
Shanely et al. [60]	48 (13 F, 35 M) trained runners	Randomized, double-blind, placebo-controlled clinical trial; 30 days (+ race day + 7 days post-marathon)	*RR*: standardized root extract (>5% rosavins, >1.8% salidroside); 600 mg/day Running (marathon race)	↔ Race performance, vertical jump, DOMS, CK, inflammatory markers, eHSP72	*RR* did not influence exercise performance, or recovery-related outcomes following marathon running
Wang et al. [63]	48 M professional basketball players	Randomized, double-blind, controlled clinical trial; 4 weeks	*RR*: capsules (0.5 g salidroside/100 g raw material); 2.4 g/day (30 min before breakfast and lunch) Simulated basketball game	↓ 5 km running time ↑ Yo-Yo test ↑ VO_2max_ ↓ RPE ↑ CMJ ↓ Post-exercise HR ↑ Total antioxidant capacity	*RR* improved aerobic capacity, reduced perceived fatigue and enhanced simulated game performance in basketball players
Dou et al. [62]	24 M competitive football players	Randomized, double-blind, placebo-controlled clinical trial; 4 weeks	*RR*: standardized capsules (salidroside: 12 mg/day); 2.4 g/day Regular football training	↑ Yo-Yo IR2 ↓ RSA mean sprint time ↓ Blood lactate ↑ Decision-making accuracy ↑ Passing performance	*RR* improved intermittent endurance and preserved cognitive and technical performance under fatigue in competitive football players

F: female; M: male; *RR*: *Rhodiola rosea*; CK: creatine kinase; TAC: total antioxidant capacity; SOD: superoxide dismutase; VO_2max_: maximal oxygen uptake; HR: heart rate; RPE: rating of perceived exertion; TTE: time-to-exhaustion; DOMS: delayed onset muscle soreness; eHSP72: extracellular heat shock protein 72; CMJ: countermovement jump; RSA: repeated sprint ability; Yo-Yo IR2: Yo-Yo Intermittent Recovery Test Level 2; ↑/↓: statistically significant higher/lower values or changes in the supplementation group versus placebo/control; ↔: no statistically significant between-group effect. Arrows indicate direction, not benefit.

**Table 4:** Effects of *Cordyceps* species supplementation on exercise recovery, exercise performance, and selected physiological responses in athletes.

Authors	Study Group	Study Design	Intervention	Outcome	Conclusion
Parcell et al. [75]	22 M endurance-trained cyclists	Randomized, double-blind, placebo-controlled clinical trial; 5 weeks	*CS*: CordyMax Cs-4^®^ tablets; 3.15 g/day; Incremental cycling test + 30 km time trial	↔ VO_2_peak, VT, Time-trial performance	*CS* did not improve aerobic capacity or endurance performance
Thongsawang et al. [76]	12 M trained long-distance runners	Randomized, single-blind, crossover clinical trial; 2 weeks	*CS*: powder; 3 g/day; Incremental treadmill test to exhaustion	↑ VO_2max_ ↑ TTE ↑ VT2 ↔ VT1	CS improved endurance performance and VO_2max_
Nakamura et al. [77]	22 M trained long-distance runners	Randomized, double-blind, placebo-controlled, parallel-group clinical trial; 16 weeks	*CM*: mycelium extract (patented strain KT165514); 1.8 g/day Pre-season endurance training; 5000 m running performance test	↑ Ferritin ↑ Hb ↑ Hct ↓ CK ↔ 5000 m performance	*CM* attenuated exercise-induced decline in iron stores and reduced muscle damage markers without improving running performance
Thongsawang et al. [78]	39 F trained soccer players	Randomized, single-blind, placebo-controlled clinical trial; 4 weeks	*CS*: powder; 3 g/day Repeated-sprint training in hypoxia or normoxia	↑ TTE ↑ Peak power ↑ Mean power ↓ Fatigue index ↓ Blood lactate ↔ VO_2max_ ↔ Hb, Hct, RBC	*CS* improved repeated-sprint performance and time to exhaustion, particularly when combined with hypoxic training, without affecting hematological variables

F: female; M: male; *CS*: *Cordyceps sinensis; CM*: *Cordyceps militaris;* VO_2_peak: peak oxygen uptake; VO_2max_: maximal oxygen uptake; TTE: time to exhaustion; VT: ventilatory threshold; VT1: first ventilatory threshold; VT2: second ventilatory threshold; Hb: hemoglobin; Hct: hematocrit; CK: creatine kinase; RBC: red blood cells; ↑/↓: statistically significant higher/lower values or changes in the supplementation group versus placebo/control; ↔: no statistically significant between-group effect. Arrows indicate direction, not benefit.

**Table 5:** Practical considerations and current evidence regarding the use of selected adaptogens in athletes.

Adaptogen	Potential Application in Athletes	Practical Considerations	Current Evidence	Main Limitations
*Withania somnifera*	Recovery, stress management, sleep support	Standardized root extracts may be considered during periods of intensified training; the extract type, withanolide content, and product quality should be verified	Relatively stronger but still limited and outcome-specific evidence, based on several small RCTs in athletic populations	Small samples, short interventions, heterogeneous extracts and outcomes, and limited replication across sports and sexes
*Panax* species	Fatigue management, endurance support	Effects may vary depending on the *Panax* species, extract standardization, and ginsenoside profile	Limited and outcome-specific evidence, with inconsistent findings across outcomes	Few athlete studies, heterogeneous *Panax* species and extracts, and variable supplementation protocols
*Rhodiola rosea*	Fatigue resistance, perceived exertion	Standardized extracts with defined rosavin and/or salidroside content should be preferred	Limited and outcome-specific evidence, with inconsistent findings across different outcomes	Heterogeneous extracts, supplementation protocols, exercise models, and outcome measures
*Cordyceps* species	Endurance performance and fatigue resistance	Species, cultivation methods, and product standardization should be considered	Preliminary evidence based on a limited number of athlete studies	Few athlete studies and heterogeneous *Cordyceps* species, supplementation protocols, and exercise models

## Data Availability

No new data were created or analyzed in this study. Data sharing is not applicable to this article.
